# Pancreaticoduodenal Artery Aneurysms Due to Median Arcuate Ligament Syndrome (MALS) Treated by Laparoscopic Dissection and Embolization: A Case Report

**DOI:** 10.7759/cureus.109137

**Published:** 2026-05-18

**Authors:** Mika Ando, Takashi Oshiro, Hidemasa Kikuchi, Shusuke Kasuya, Shiniichi Okazumi

**Affiliations:** 1 Surgery, Tohoku University, Sendai, JPN; 2 Surgery, Toho University Medical Center Sakura Hospital, Sakura, JPN; 3 Internal Medicine, Toho University Medical Center Sakura Hospital, Sakura, JPN; 4 Radiology, Toho University Medical Center Sakura Hospital, Sakura, JPN

**Keywords:** aneurysm rupture, artery aneurysm rupture, laparoscopic surgery, median arcuate ligament syndrome, pancreatic arcuate aneurysm, transcatheter arterial embolization

## Abstract

Pancreatic arcade aneurysms are uncommon vascular lesions but may be fatal when rupture occurs. We describe the case of a 68-year-old woman who presented with hematemesis and profound anemia and was diagnosed with multiple pancreaticoduodenal artery aneurysms associated with median arcuate ligament syndrome (MALS). Contrast-enhanced computed tomography (CT) revealed a severe stenosis of the celiac artery origin with post-stenotic dilatation and aneurysmal changes within the pancreatic arterial arcade. Active bleeding from a ruptured posterior superior pancreaticoduodenal artery (PSPDA) aneurysm was successfully controlled by emergency coil embolization. To address the underlying hemodynamic abnormality and reduce the risk of further aneurysm formation or rupture, elective laparoscopic median arcuate ligament release was subsequently performed. Intraoperative Doppler ultrasonography demonstrated the restoration of antegrade hepatic arterial flow following decompression. This case highlights the importance of combining endovascular and surgical approaches in the management of pancreatic arcade aneurysms associated with MALS, with careful long-term follow-up.

## Introduction

Pancreatic arcade aneurysms are rare vascular lesions. Median arcuate ligament syndrome (MALS) is characterized by the extrinsic compression of the celiac artery, leading to stenosis or occlusion and the subsequent redistribution of blood flow from the superior mesenteric artery (SMA) through the pancreatic arterial arcade [1-3]. The median arcuate ligament passes superior to the origin of the celiac artery and may compress it, resulting in stenosis. This leads to increased collateral flow from the superior mesenteric artery (SMA), which functions as a compensatory pathway, potentially causing aneurysm formation due to increased hemodynamic stress. In this setting, the superior mesenteric artery acts as a compensatory “backup” source of blood flow. MALS is a rare condition caused by the external compression of the celiac artery by the median arcuate ligament, leading to altered splanchnic hemodynamics. Although it may present with postprandial abdominal pain, weight loss, or nausea, many patients remain asymptomatic due to well-developed collateral circulation.

This altered hemodynamic state results in increased collateral flow through the pancreaticoduodenal arterial arcade and generates abnormal hemodynamic stress, which is considered a key mechanism underlying aneurysm development and progression in patients with MALS [4-7]. Imaging modalities such as computed tomography (CT) and angiography are essential for the diagnosis and exclusion of other etiologies, and visceral artery aneurysms are recognized as an important secondary finding in MALS [8,9].

Pancreatic arcade aneurysms, including aneurysms of the pancreaticoduodenal arteries, are clinically significant because rupture can result in life-threatening retroperitoneal or gastrointestinal hemorrhage [10-13]. In the management of ruptured pancreatic arcade aneurysms, endovascular treatment has become the preferred initial approach because it enables rapid and minimally invasive hemorrhage control [11-14]. However, when celiac artery compression persists after embolization, excessive collateral flow may remain, predisposing patients to the rupture of residual aneurysms or the formation of new visceral artery aneurysms [15].

Accordingly, the surgical release of the median arcuate ligament has been advocated to correct the underlying hemodynamic disturbance and reduce long-term vascular risk [16,17]. Herein, we report a case of multiple pancreaticoduodenal artery aneurysms associated with MALS, including a ruptured lesion causing active bleeding, which was treated by emergency coil embolization, followed by elective laparoscopic median arcuate ligament release.

## Case presentation

A 68-year-old woman presented to the emergency department with dark red hematemesis that had begun approximately 12 hours before admission. She had a medical history of hypertension, breast cancer treated by mastectomy at the age of 59 years, and ovarian cancer treated by total abdominal hysterectomy with bilateral salpingo-oophorectomy and pelvic lymphadenectomy at the age of 66 years. No evidence of celiac artery stenosis had been reported on prior imaging performed for ovarian cancer surveillance. There was no relevant family history. There was no history of recent weight loss prior to presentation.

On presentation, the patient was alert and oriented but appeared markedly pale. Her vital signs showed a temperature of 36.8°C, blood pressure of 98/61 mmHg, pulse rate of 108 beats per minute, and respiratory rate of 18 breaths per minute. Oxygen saturation was 85% on room air and improved to 100% with supplemental oxygen at 3 L/minute via nasal cannula. Physical examination revealed a pale conjunctiva but was otherwise unremarkable, with no abdominal tenderness. Her body mass index (BMI) was 30.7 kg/m².

Laboratory investigations demonstrated severe anemia, with a hemoglobin level of 3.7 g/dL (Table 1). The white blood cell count was elevated to 13,300/µL, while the C-reactive protein level was within the normal range at 0.12 mg/dL. Blood urea nitrogen was elevated at 46.7 mg/dL with a creatinine level of 0.66 mg/dL, suggesting dehydration secondary to acute blood loss.

**Table 1:** Laboratory findings BUN: blood urea nitrogen

Laboratory parameter	Measured value	Normal range
Hemoglobin (g/dL)	3.7	11.5-15.0
C-reactive protein (mg/dL)	0.12	<0.30
White blood cell count (/µL)	13,300	3,300-8,600
Blood urea nitrogen (mg/dL)	46.7	8-20
Creatinine (mg/dL)	0.66	0.65-1.07
BUN/creatinine ratio	70.8	-

Contrast-enhanced abdominal computed tomography

Contrast-enhanced computed tomography (CT) of the abdomen showed marked stenosis at the origin of the celiac artery with post-stenotic dilatation (Figure 1). Two aneurysms were found in the posterior superior pancreaticoduodenal artery (PSPDA), one of which showed extravasation. Replaced right hepatic artery (RHA) originating from the superior mesenteric artery (SMA) and one aneurysm were also observed at its bifurcation (Figure 2).

**Figure 1:**
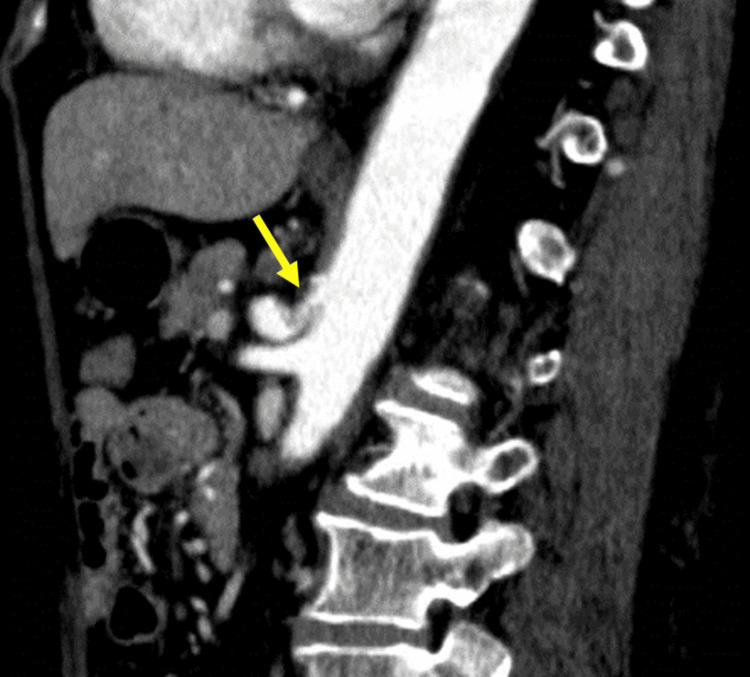
Sagittal view CT shows stenosis and the post-stenotic dilatation of the origin of the celiac artery (yellow arrow) CT: computed tomography

**Figure 2:**
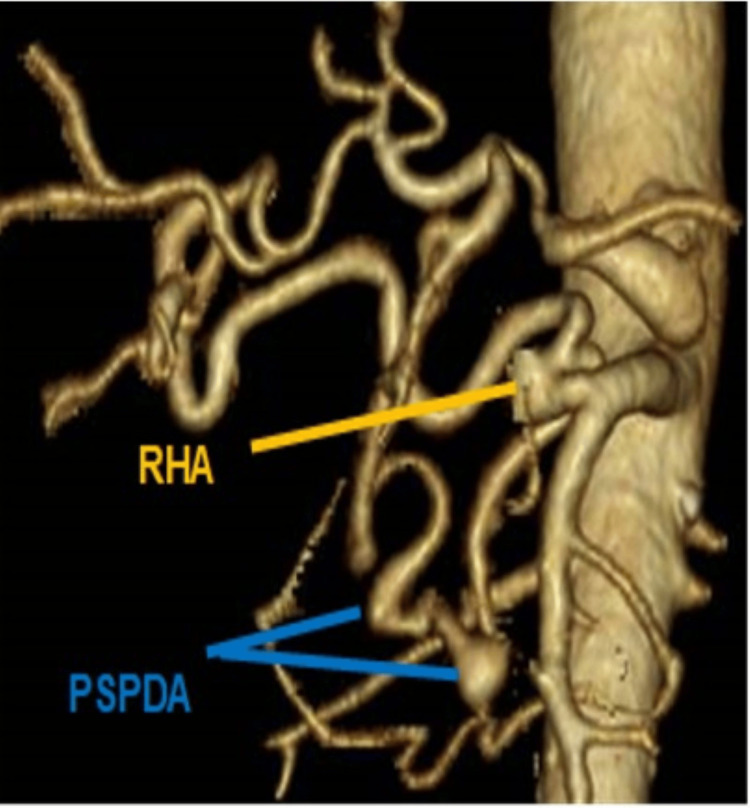
Three-dimensional CT Aneurysms of the posterior superior pancreaticoduodenal artery (PSPDA) and right hepatic artery (RHA) are noted CT: computed tomography

Angiographic findings

SMA angiography revealed the left hepatic artery supplied through the pancreatic arterial arcade and a replaced right hepatic artery (Figure 3). Contrast medium extravasation was observed from one of the posterior superior pancreaticoduodenal artery aneurysms.

**Figure 3:**
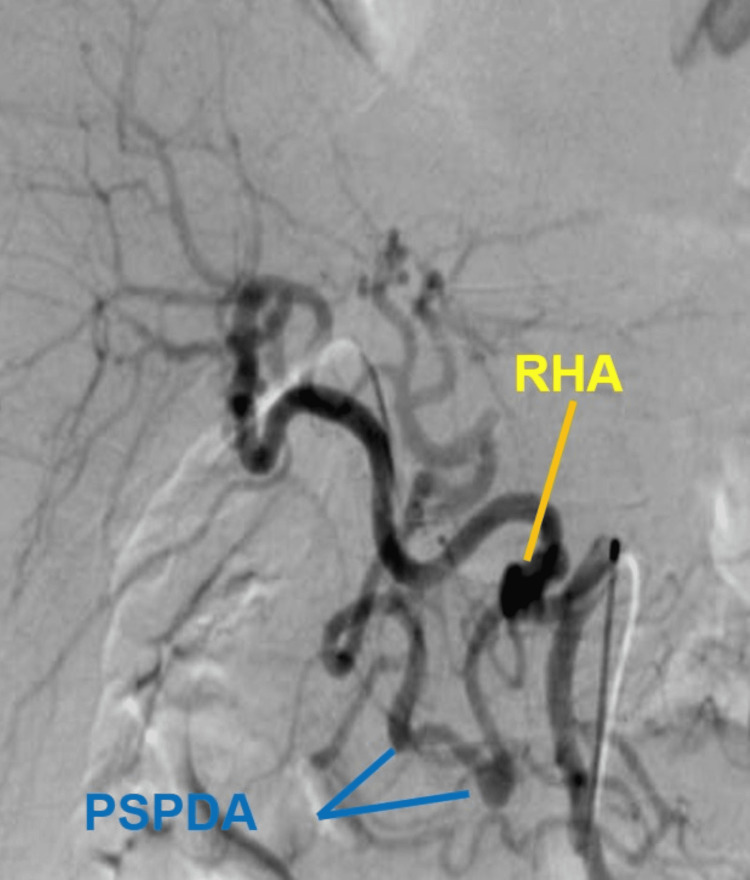
SMA angiography identifying the right hepatic artery (RHA) and the posterior superior pancreaticoduodenal artery (PSPDA) with aneurysms SMA: superior mesenteric artery

Angioembolization

SMA arteriography showed the left hepatic artery to be supplied by the gastroduodenal artery (GDA) through a well-developed pancreatic arcade against the background of the stenotic proximal celiac artery.

Angiography/angioembolization procedures were performed using an Excelsior 1018® microcatheter (Stryker, Kalamazoo, MI) and a Meister® 0.016-inch guidewire (Asahi Intecc, Seto, Japan). PSPDA angiography showed the extravasation (Figure 4), and we decided to embolize both PSPDA aneurysms in a retrograde fashion, starting at the extravasation point. Six coils were used for flaming, and 10 coils were used for filling. Confirmatory angiography performed from the celiac artery to the GDA revealed that the coils did not deviate from the main trunk of the GDA and that the extravasation had been eliminated (Figure 5).

**Figure 4:**
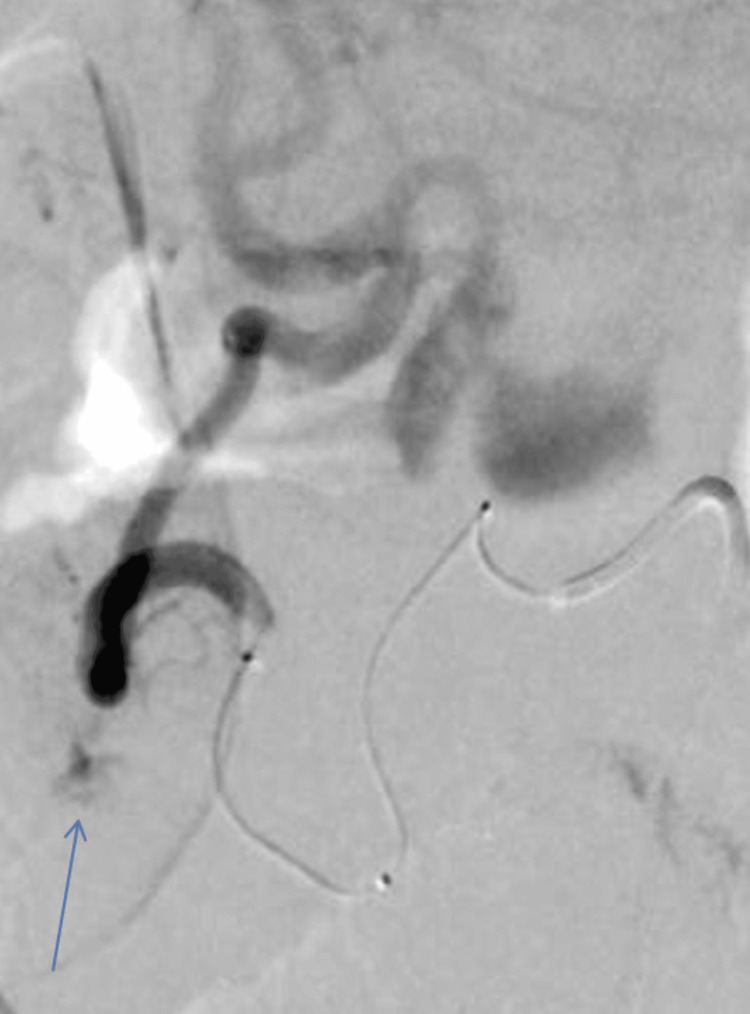
Contrast extravasation from one of two posterior superior pancreaticoduodenal arteries (PSPDA)

**Figure 5:**
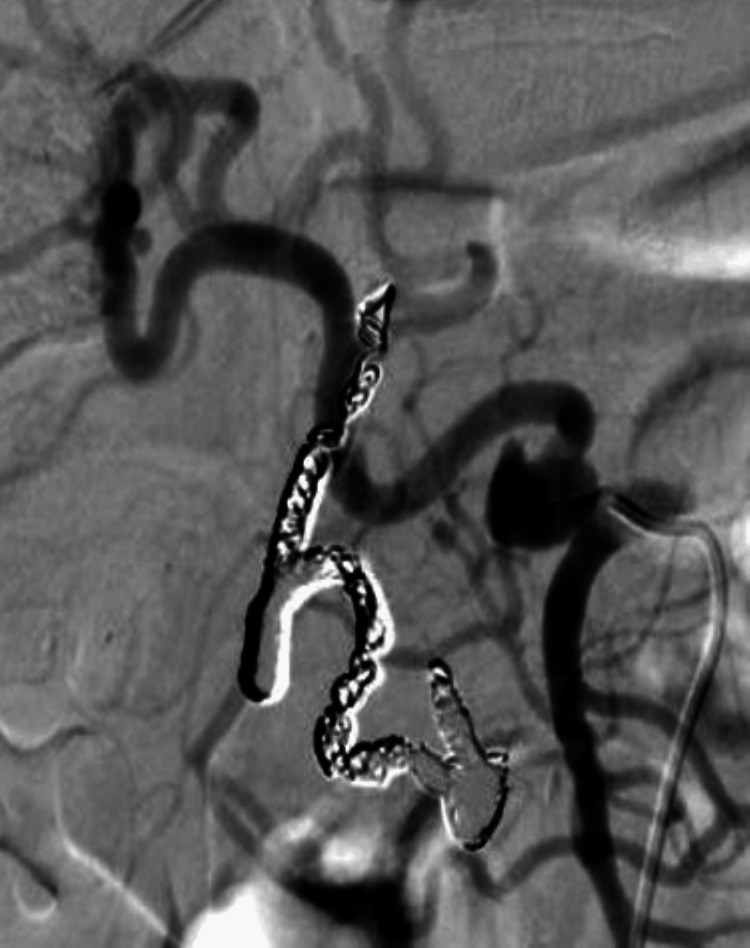
After PSPDA coil embolization Showing the complete cessation of bleeding PSPDA: posterior superior pancreaticoduodenal artery

Surgical findings

In the absence of any other obvious etiology, MALS was thought to be a factor in the formation and expansion of the visceral aneurysms. A laparoscopic decompression procedure for MALS was performed two months after interventional radiology (IVR), the interval allowed for hemodynamic stabilization and careful surgical planning.

The operation was performed under general anesthesia in the lithotomy position. A total of five ports were used. A 12 mm camera port was placed 3 cm above the umbilicus. Two 5 mm ports were inserted in the right and left subcostal regions along the anterior axillary lines. Two additional 12 mm ports were placed in the right and left lateral abdomen; the right-sided port was positioned slightly medial to the midclavicular line. The procedure was performed with one assistant, who managed the retraction of the liver and stomach while the operator performed the dissection. The flexible scope was routinely used in our institution and provided improved visualization of the deep operative field, particularly around the celiac axis.

The lateral segment of the left liver was elevated using a silicone disk and a snake retractor inserted through the right lateral port.

The lesser omentum and the peritoneum anterior to the right diaphragmatic crus were incised, the anterior crus was dissected, and the left diaphragmatic crus was exposed. The esophagus was gently retracted to improve exposure without the need for extensive mobilization. The omental bursa was then opened, and the left gastric artery was suspended with tape. The left gastric artery was retracted with cloth tape, and the median arcuate ligament was dissected toward the root of the celiac artery (Figure 6). Intraoperative Doppler ultrasonography was performed using a laparoscopic Doppler probe to evaluate blood flow in the common hepatic artery (Table 2). Preoperative ultrasonography demonstrated equivocal flow direction in the splenic artery (Figure 7). In contrast, intraoperative Doppler ultrasonography confirmed the restoration of antegrade arterial flow after the release of the median arcuate ligament (Figure 8).

**Figure 6:**
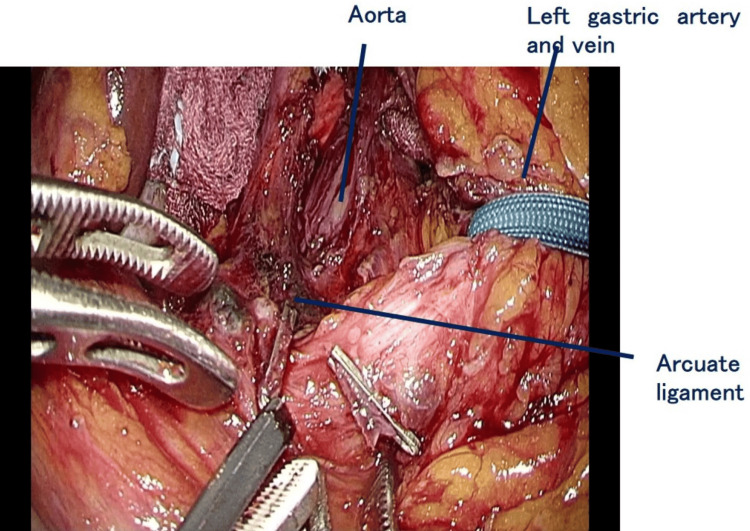
The left gastric artery was retracted with cloth tape The arcuate ligament was dissected toward the root of the celiac artery

**Table 2:** Evaluation of arterial blood flow before and after arterial ligament dissection

Measurement site/parameter	Preoperative	Intraoperative	Postoperative
Origin of the celiac artery (peak systolic velocity, cm/second)	2.9 cm/second	-	307 cm/second
Common hepatic artery (peak systolic velocity, cm/second)	20 cm/second (turbulent)	200 cm/second (antegrade flow)	138 cm/second (antegrade flow)

**Figure 7:**
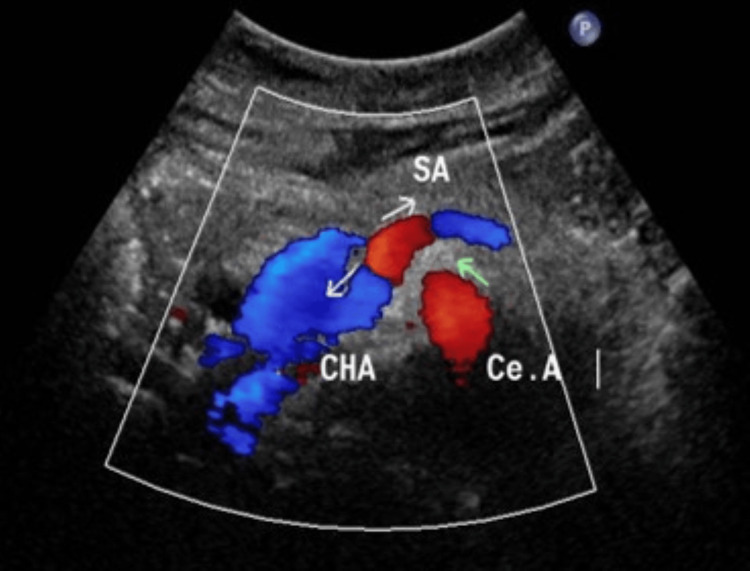
Preoperative ultrasound image showing an equivocal direction of flow in the splenic artery CHA, common hepatic artery; SA, splenic artery; Ce.A, celiac artery

**Figure 8:**
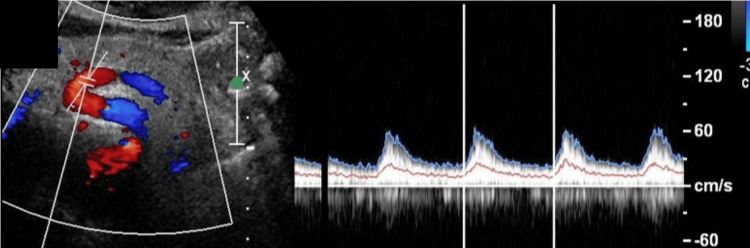
Intraoperative ultrasound image showing the restoration of flow

The muscle fibers and ligaments were separated at the intersection of the left and right diaphragmatic crura, and the anterior surface of the aorta was exposed. The surgical field was developed by further retracting the left gastric artery with the tape. The fibrous tissue around the left gastric artery extending to the root of the celiac artery was dissected using a laparoscopic coagulation shear (LCS).

Doppler ultrasonography was performed again to confirm the improvement of blood flow in the common hepatic artery (Figure 8 and Table 2). The right and left diaphragmatic crura were sutured with two stitches of 2-0 nonabsorbable polyester sutures. Operative time was four hours and 51 minutes, and blood loss was 10 mL.

Postoperative course

Delayed gastric emptying was observed on the second postoperative day, requiring the insertion of a gastric tube. The patient started eating on the 10th postoperative day and was discharged on the 13th postoperative day with no further problems.

Contrast-enhanced CT performed one month postoperatively demonstrated persistent but stable stenosis of the celiac artery (Figure 9). Furthermore, contrast-enhanced magnetic resonance imaging (MRI) obtained 10 months after surgery confirmed that the aneurysm at the bifurcation of the right hepatic artery remained unchanged and that no new aneurysms had developed (Figure 10).

**Figure 9:**
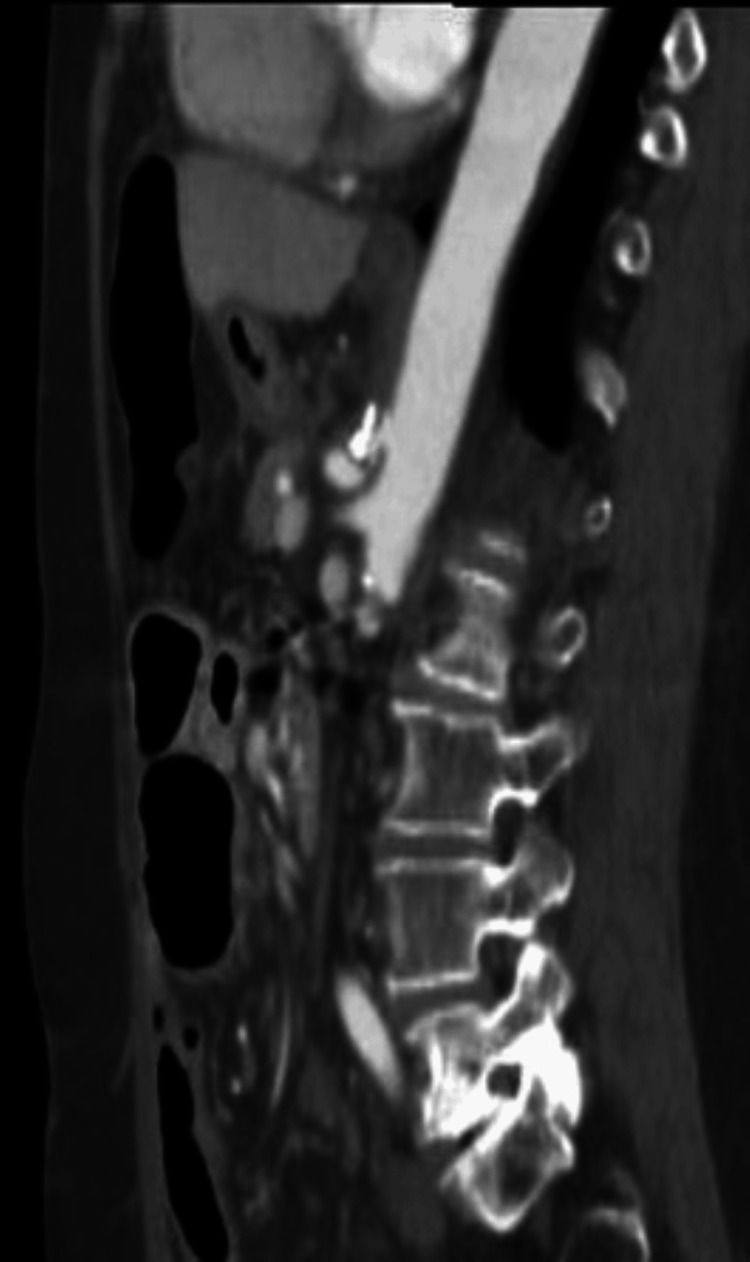
Contrast-enhanced CT one month after surgery CT: computed tomography

**Figure 10:**
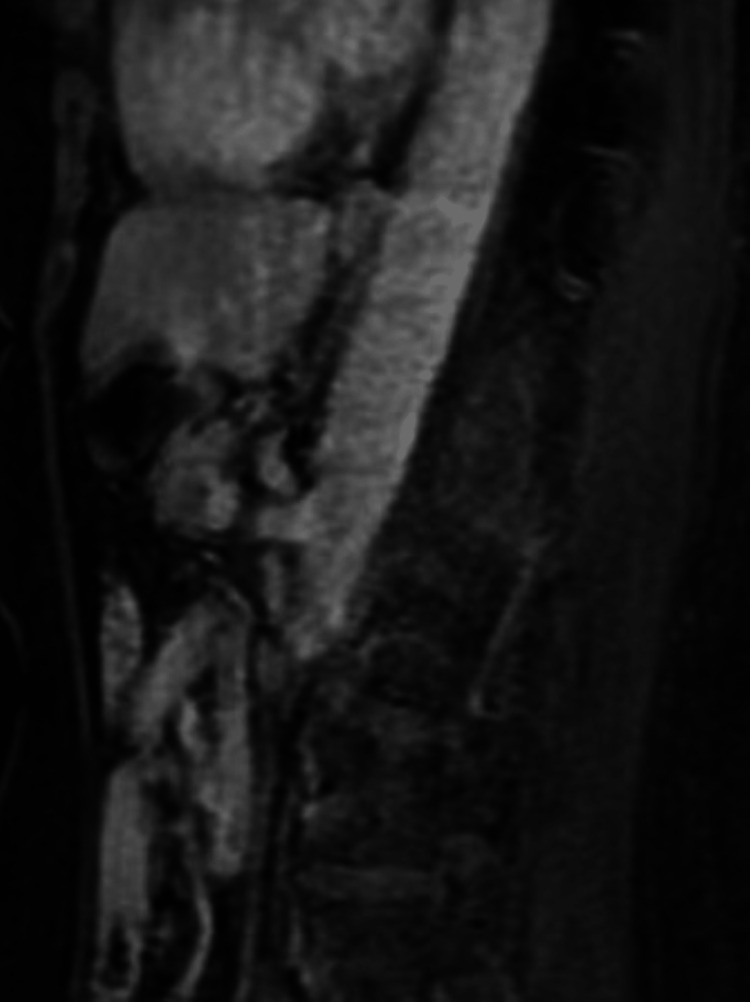
Contrast-enhanced MRI 10 months after surgery MRI: magnetic resonance imaging

## Discussion

This case illustrates the pathophysiological relationship between MALS and pancreaticoduodenal artery aneurysm formation. The clinical presentation reflects the consequences of aneurysmal rupture rather than the underlying MALS itself, and definitive diagnosis relies on imaging modalities such as contrast-enhanced CT and angiography (Figure 11).

**Figure 11:**
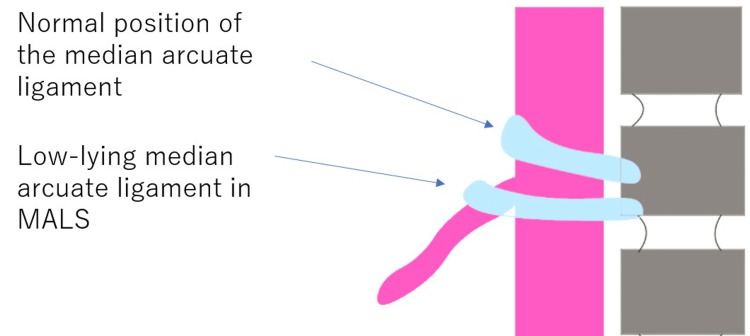
Pathophysiological mechanism of MALS Under normal conditions, the median arcuate ligament passes superior to the origin of the celiac artery without causing compression, thereby maintaining normal antegrade blood flow through the celiac axis. The celiac plexus is also not compressed, and the physiological perfusion of the upper abdominal organs is preserved. However, in MALS, the median arcuate ligament compresses the origin of the celiac artery, resulting in stenosis and reduced antegrade blood flow. Consequently, collateral circulation from the superior mesenteric artery (SMA) through the pancreaticoduodenal arcade, including the inferior pancreaticoduodenal artery (IPDA), becomes increased. The resulting hemodynamic stress may contribute to aneurysm formation. In addition, the compression of the celiac plexus may lead to neuropathic symptoms MALS: median arcuate ligament syndrome

In 1917, Lipshutz reported the first case of the anatomical compression of the celiac artery by the crus diaphragm [1]. In 1963, Harjola first reported the “celiac axis compression syndrome” [2]. The most common source of compression is the median arcuate ligament (where the right and left crura of the diaphragm join at the anterior surface of the vertebral body) in 63% of cases, but other causes include cancer invasion, atherosclerotic changes, celiac plexus thickening, and scar tissue [3].

The characteristic definitive imaging findings of MALS include a focal narrowing with a hooked appearance and respiratory variation in the degree of stenosis, which can also be evaluated using ultrasound imaging [4].

MALS is caused by a stenosis of the celiac artery when it bifurcates more cephalad than usual or when the median arch ligament of the diaphragm is deviated caudally, even if the origin of the bifurcation is normal, and it is well-documented that this condition can lead to hemodynamic stress and visceral artery complications [5]. Consequently, several clinical reports have specifically associated this anatomical compression with the development of pancreaticoduodenal artery aneurysms [6]. The celiac artery moves caudally during inspiration, reducing the stenosis at its origin, but during expiration, the celiac artery becomes more stenotic, and blood flow out of the liver is increased [4].

MALS is more common in women and can be associated with postprandial ischemic symptoms (abdominal pain and vomiting) but is often asymptomatic due to the development of collateral circulation. Although MALS is more commonly reported in younger individuals, particularly women under 40 years of age, our patient did not have recent weight loss or other precipitating factors. It is possible that anatomical predisposition or previously unrecognized compression contributed to the delayed presentation in this case.

The diagnosis is confirmed when abdominal aortography shows an indentation-like stenosis at the origin of the celiac artery from the lateral side. Characteristic imaging findings include a focal narrowing with a hooked appearance and respiratory variation in the degree of stenosis. Although dynamic imaging was limited in this case, the presence of collateral circulation supported the diagnosis. CT is necessary to exclude other etiologies, such as atherosclerosis. As a secondary finding in MALS, the formation of visceral aneurysms may be observed [7,8]. Because blood flow that should normally be directed from the celiac artery to the common hepatic artery and left gastric artery is reduced in MALS, blood flow is supplied to the liver and spleen from the SMA through the pancreaticoduodenal artery arcade. Increased retrograde blood flow in the arteries of this arcade has been reported to cause aneurysm formation due to hemodynamic stress [7-9].

The prevalence of abdominal visceral aneurysms in the general population is said to be approximately 1%, and with the widespread use of CT, more and more asymptomatic cases are being detected [8-10]. Among visceral aneurysms, splenic artery aneurysms are the most common at 60%, followed by hepatic artery aneurysms at 20%, while aneurysms of the SMA, celiac artery, and GDA are reported to occur in a few percent of cases [7,8]. Pancreatic arcade aneurysms are rare, occurring in about 2%, but about 30% of these aneurysms are associated with MALS [10-12].

Pancreatic arcade aneurysms have a high mortality rate of 8.5%-23% when detected following rupture or if rupture occurs during follow-up [11-13]. There is no correlation between aneurysm rupture and aneurysm diameter [11,12].

The possibility of rupture, regardless of aneurysm diameter, should be taken into account to allow the opportunity for therapeutic intervention.

Pancreatic arcade aneurysms are located in a surgically awkward area. Treatment is usually instituted to prevent life-threatening hemorrhagic shock owing to aneurysmal rupture bleeding. In the treatment of pancreatic arcade aneurysms with a stenosis of the origin of the celiac artery, IVR has been used for 23 of 31 cases (74.2%) since 2000 [11]. Surgical treatment is chosen when transcatheter embolization is difficult or unsuccessful. Although inflow artery ligation and aneurysmectomy are often selected as surgical techniques, the mortality rate of surgical treatment has been reported to be 23%-29.4% [11,14]. Surgical complications include retroperitoneal hematoma formation and pancreatitis.

Endovascular balloon dilatation and stenting have also been used to relieve the celiac artery stenosis caused by the compression of the arcuate ligament, which is the underlying cause of pancreatic arcade aneurysms, but the long-term results are unknown. There have been reports of ongoing patency for approximately two years after surgery [8,15]. There are also reports of aneurysm remodeling following balloon dilatation and stenting, although restenosis may occur [8,15].

In this case of MALS, the patient was found to have a ruptured aneurysm in the pancreatic arcade and was treated urgently with IVR to achieve hemostasis, but the aneurysm at the origin of the RHA was not treated with IVR to maintain hepatic blood flow. Considering the possibility of the rupture of the remaining aneurysm or the development of aneurysms at other sites due to hemodynamic changes caused by MALS even after IVR, we decided on an arch ligament dissection, followed by a wait-and-see policy. The residual right hepatic artery aneurysm was managed conservatively because it remained stable, and the correction of the underlying hemodynamic abnormality was expected to reduce rupture risk. Careful imaging follow-up was planned.

Nojima et al. reported that up to 2012, 13 of 14 patients who had undergone arch ligament dissection for MALS had had a restoration of blood flow with ligament dissection alone (one patient did not have restoration and underwent bypass from the left renal artery to the splenic artery) [16].

In recent years, the laparoscopic dissection of the median arcuate ligament has also been reported, although surgical cases performed after aneurysm rupture remain limited. A literature search using the terms “median arcuate ligament syndrome,” “laparoscopy,” and “aneurysm rupture” identified three previously reported cases of laparoscopic median arcuate ligament release performed after aneurysm rupture, in addition to the present case (Table 3). No intraoperative complications were reported in these cases, and postoperative hospital stays were generally short.

**Table 3:** Case studies PSPDA, posterior superior pancreaticoduodenal artery; RHA, right hepatic artery; M, male; F, female; IVR, interventional radiology

Author	Year	Age	Sex	Complaint	Aneurysm location	Postoperative hospital stay (days)	Period from IVR to operation	Complications
Hanaki et al. [18]	2018	47	M	Sudden epigastric pain	Pancreaticoduodenal artery (PDA)	8	3 months	None
Tokuda et al. [19]	2019	66	F	Acute abdominal pain	Inferior pancreaticoduodenal artery (IPDA)	22	Same time	None
Tokuda et al. [19]	2019	75	M	Acute abdominal pain, nausea, and cold sweat	PDA	-	-	None
Our case	2022	68	F	Hematemesis	PSPDA and RHA	13	2 months	None

Jimenez et al. also reported the results of open or laparoscopic surgical intervention for MALS of 400 patients; 279 underwent open surgery, and 121 underwent laparoscopic surgery [17]. The follow-up period ranged from 10 to 229 months in the open group and six to 44 months in the laparoscopic group. Overall, 85% of the patients had immediate postoperative relief (78% in the open group and 96% in the laparoscopic group). Late recurrence was reported in 19 patients (6.8%) in the open group and in seven patients (5.7%) in the laparoscopic group. They noted that no procedure-related or early perioperative deaths were reported, although the quality of life and adverse event data were not compared.

Laparoscopic surgery in this region may be associated with technical challenges, including limited working space and the need for careful dissection around major vascular structures. However, these intraoperative issues can be effectively managed using modern ultrasonic energy devices and advanced laparoscopic techniques. Additionally, some degree of minor oozing is commonly encountered during dissection in the retroperitoneal region, including procedures such as MALS release or lymphadenectomy, and can generally be controlled without difficulty.

## Conclusions

This case underscores the importance of a multidisciplinary treatment strategy for pancreaticoduodenal artery aneurysms associated with median arcuate ligament syndrome (MALS). Endovascular therapy is essential for the rapid and effective control of life-threatening hemorrhage due to aneurysmal rupture. However, definitive management requires the correction of the underlying hemodynamic abnormality through median arcuate ligament release to prevent the recurrence or formation of new aneurysms. In addition, given the unpredictable rupture risk of visceral artery aneurysms, careful long-term surveillance with serial imaging is crucial. This case highlights the complementary roles of endovascular and surgical approaches in achieving both immediate and durable outcomes.

## References

[1] Lipshutz B (1917). A composite study of the coeliac axis artery. Ann Surg.

[2] Harjola PT (1963). A rare obstruction of the coeliac artery. Report of a case. Ann Chir Gynaecol Fenn.

[3] Ueno S, Maemura K, Sakoda M (2011). Release of median-arcuate ligament under laparotomy for patients with hepatobiliary-pancreatic cancer complicated with celiac artery compression syndrome. Med J Kagoshima Univ.

[4] Waki H, Akamatsu H, Tori M, Nakahara M (2009). Ultrasound image of compression syndrome at the origin of the celiac artery. Mod Physician.

[5] Fukuda A, Yamamoto K, Matsuda H, Matsuura H (2003). A case of inferior pancreatic duodenal artery aneurysm associated with median arcuate ligament compression syndrome (Article in Japanese). J Jpn Soc Clin Surg.

[6] Yamaguchi M, Tokumaru T, Nagamine N, Kai H (2010). A case of the pancreaticoduodenal aneurysm due to the median arcuate ligament compression syndrome (Article in Japanese). J Jpn Soc Clin Surg.

[7] Goodall R, Langridge B, Onida S, Ellis M, Lane T, Davies AH (2020). Median arcuate ligament syndrome. J Vasc Surg.

[8] Tien YW, Kao HL, Wang HP (2004). Celiac artery stenting: a new strategy for patients with pancreaticoduodenal artery aneurysm associated with stenosis of the celiac artery. J Gastroenterol.

[9] Kamata S, Fujita Y, Nitta H, Ishikawa F, Ito H (2015). A case of peritoneal hematoma with celiac artery root stenosis due to median arcuate ligament compression syndrome (Article in Japanese). J Jpn Soc Clin Surg.

[10] Philip SK, Paty PS, Cordero JA, Darling RC, Chang BB, Shah DM, Leather RP (1996). Aneurysms of the pancreaticoduodenal artery. J Vasc Surg.

[11] Oishi K, Suzuki S, Sakaguchi T, Hukumoto K, Inaba K, Konno H (2009). A case of dorsal pancreatic aneurysm due to median arcuate ligament compression syndrome (Article in Japanese). J Jpn Soc Clin Surg.

[12] Corey MR, Ergul EA, Cambria RP, Patel VI, Lancaster RT, Kwolek CJ, Conrad MF (2016). The presentation and management of aneurysms of the pancreaticoduodenal arcade. J Vasc Surg.

[13] Sessa C, Tinelli G, Porcu P, Aubert A, Thony F, Magne JL (2004). Treatment of visceral artery aneurysms: description of a retrospective series of 42 aneurysms in 34 patients. Ann Vasc Surg.

[14] Sato T, Nagasaki K, Nomura Y (2013). Rupture of a pancreaticoduodenal arterial aneurysm into the duodenum complicated with celiac artery stenosis caused by compression of the median arcuate ligament (Article in Japanese). J Jpn Coll Angiol.

[15] Savastano S, Feltrin GP, Miotto D, Chiesura-Corona M, Sandri P (1995). Embolization of ruptured aneurysm of the pancreaticoduodenal artery secondary to long-standing stenosis of the celiac axis: case reports. Vascular Surgery.

[16] Nojima H, Takano S, Otsuka M, Kimura H, Shimizu H, Miyazaki M (2012). A case of successful pancreaticoduodenectomy in a patient with celiac artery compression syndrome: effectiveness of hepatic arterial flow monitoring (Article in Japanese). J Jpn Surg Assoc.

[17] Jimenez JC, Harlander-Locke M, Dutson EP (2012). Open and laparoscopic treatment of median arcuate ligament syndrome. J Vasc Surg.

[18] Hanaki T, Fukuta S, Okamoto M (2018). Median arcuate ligament syndrome and aneurysm in the pancreaticoduodenal artery detected by retroperitoneal hemorrhage: a case report. Clin Case Rep.

[19] Tokuda S, Sakuraba S, Orita H, Sakurada M, Kushida T, Maekawa H, Sato K (2019). Aneurysms of pancreaticoduodenal artery due to median arcuate ligament syndrome, treated by open surgery and laparoscopic surgery. Case Rep Surg.

